# Evidence that the metabolite repair enzyme NAD(P)HX epimerase has a moonlighting function

**DOI:** 10.1042/BSR20180223

**Published:** 2018-05-08

**Authors:** Thomas D. Niehaus, Mona Elbadawi-Sidhu, Lili Huang, Laurence Prunetti, Jesse F. Gregory, Valérie de Crécy-Lagard, Oliver Fiehn, Andrew D. Hanson

**Affiliations:** 1 Horticultural Sciences Department, University of Florida, Gainesville, FL, U.S.A.; 2 NIH West Coast Metabolomics Center, Genome Center, University of California Davis, Davis, CA, U.S.A.; 3 Food Science and Human Nutrition Department, University of Florida, Gainesville, FL, U.S.A.; 4 Microbiology and Cell Science Department, University of Florida, Gainesville, FL, U.S.A.

**Keywords:** AIBP, NAXE, NAD(P)H hydrates, NAD(P)HX, protein moonlighting, Vitamin B6

## Abstract

NAD(P)H-hydrate epimerase (EC 5.1.99.6) is known to help repair NAD(P)H hydrates (NAD(P)HX), which are damage products existing as *R* and *S* epimers. The *S* epimer is reconverted to NAD(P)H by a dehydratase; the epimerase facilitates epimer interconversion. Epimerase deficiency in humans causes a lethal disorder attributed to NADHX accumulation. However, bioinformatic evidence suggest caution about this attribution by predicting that the epimerase has a second function connected to vitamin B_6_ (pyridoxal 5′-phosphate and related compounds). Specifically, (i) the epimerase is fused to a B_6_ salvage enzyme in plants, (ii) epimerase genes cluster on the chromosome with B_6_-related genes in bacteria, and (iii) epimerase and B_6_-related genes are coexpressed in yeast and *Arabidopsis*. The predicted second function was explored in *Escherichia coli*, whose epimerase and dehydratase are fused and encoded by *yjeF*. The putative NAD(P)HX epimerase active site has a conserved lysine residue (K192 in *E. coli* YjeF). Changing this residue to alanine cut *in vitro* epimerase activity by ≥95% but did not affect dehydratase activity. Mutant cells carrying the K192A mutation had essentially normal NAD(P)HX dehydratase activity and NAD(P)HX levels, showing that the mutation had little impact on NAD(P)HX repair *in vivo*. However, these cells showed metabolome changes, particularly in amino acids, which exceeded those in cells lacking the entire *yjeF* gene. The K192A mutant cells also had reduced levels of ‘free’ (i.e. weakly bound or unbound) pyridoxal 5'-phosphate. These results provide circumstantial evidence that the epimerase has a metabolic function beyond NAD(P)HX repair and that this function involves vitamin B_6_.

## Introduction

**Figure 1:**
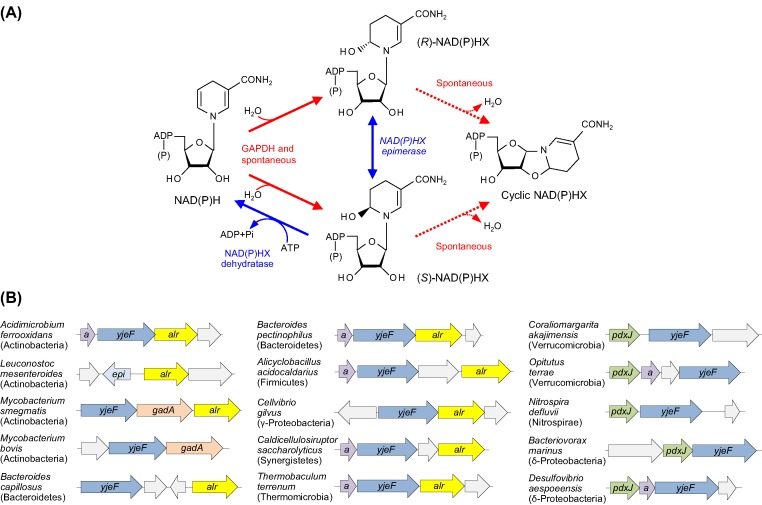
NAD(P)H damage and repair reactions, and the neighborhoods of genes encoding the repair enzymes in bacteria (**A**) Chemical and enzymatic damage reactions convert NAD(P)H to *R* and *S* forms of NAD(P)HX, which are reconverted to NAD(P)H by the repair reactions mediated by NAD(P)HX epimerase and NAD(P)HX dehydratase. Bacterial NAD(P)HX dehydratase uses ADP instead of ATP [5]. Both forms of NAD(P)HX can spontaneously cyclize. The enzymatic formation of NAD(P)HX is due to a side reaction of glyceraldehyde 3-phosphate dehydrogenase [2]. (**B**) Representative cases of clustering on bacterial chromosomes of epimerase–dehydratase fusion genes (*yjeF*) or standalone epimerase genes (*epi*) with genes predicted to encode the pyridoxal 5′-phosphate (PLP)-dependent enzymes alanine racemase (*alr*) and glutamate decarboxylase (*gadA*), or the PLP biosynthesis enzyme pyridoxine 5'-phosphate synthase (*pdxJ*). Clustering in seven phyla with holo-acyl carrier protein synthase genes (*a*) is also shown; its significance is unclear but may relate to the major consumption of NADPH in lipid synthesis. Genes with functions that are unknown or unrelated to NAD(P)H or PLP are colored gray. Phyla to which species belong are shown in parentheses.

Damaging enzymatic and chemical side reactions in all organisms convert NADH and NADPH to their hydrates, NADHX and NADPHX, which exist as *R* and *S* epimers [1,2]. The hydrates, which inhibit various dehydrogenases [3,4], are reconverted to NAD(P)H by the sequential actions of NADP(H)X epimerase (EC 5.1.99.6) and NAD(P)HX dehydratase (EC 4.2.1.93) [5–7] (Figure 1A). Both enzymes occur in all domains of life [5]. The dehydratase is specific for the *S* form of NAD(P)HX [1] and the epimerase facilitates conversion of the *R* form to the *S* form used by the dehydratase [5–7]. Over time, if not reconverted to NAD(P)H, the *R* and *S* forms of NAD(P)HX give rise spontaneously to cyclic forms of NAD(P)HX, which are not substrates for the epimerase or the dehydratase [1,5] (Figure 1A). Together, the formation and removal of NAD(P)HX constitute an archetypal example of metabolite damage and repair [8–10]. The functions of the epimerase and the dehydratase are supported by biochemical evidence from mammals, yeast, *Escherichia coli*, and plants [5–7], and that of the dehydratase is also supported by genetic and metabolomic evidence from *Arabidopsis* [6].

The *in vivo* function of the NAD(P)HX dehydratase protein seems to match its *in vitro* enzymatic activity [6], but the situation for the epimerase protein may be more complex, as several discordant observations suggest. First, as NAD(P)HX epimers equilibrate spontaneously and quite rapidly (*t*_½_ = 40 min) in physiological conditions, it is not entirely clear why epimerase activity is needed [1]. Second, while the epimerase is fused to the dehydratase in most prokaryotes, it is fused in plants to pyridoxine/pyridoxamine phosphate oxidase (PPOX), a salvage enzyme for vitamin B_6_, i.e. pyridoxal 5′-phosphate (PLP) and related compounds [5–7,11,12]. Since protein fusions imply functional relationships between the partners [13,14], the PPOX fusion points to a B_6_ connection, in plants at least. Third, and relatedly, epimerase and dehydratase expression are not correlated in *Arabidopsis* [7]. Fourth, the mammalian epimerase (NAXE) occurs in extracellular compartments (cerebrospinal fluid, urine, and the plasma of septic patients) [15], which the dehydratase does not—although both enzymes occur in the mitochondria and cytosol [16]. Moreover, NAXE can bind apolipoprotein A-1, a plasma protein that is a major component of the high-density lipoprotein complex [15]. This binding is proposed to link NAXE to cholesterol transport, atherosclerosis, and angiogenesis [17,18].

To summarize the above, there are reasons to suspect that NAD(P)HX epimerase is more than just an epimerase, and that it has a second (i.e. moonlighting) function [19] related to B_6_. This suspicion has become medically relevant with the discovery that nonsense or missense mutations in the human epimerase gene *NAXE* (also called *AIBP*) lead to a neurodegenerative disease that is lethal in infancy [20, 21]. The etiology of this disease–and potential therapies—has so far been conceived solely in terms of loss of NAD(P)HX epimerase activity [20,21]. While the NAXE protein deficiency and cyclic NADHX accumulation observed in fibroblasts from affected individuals [21] are consistent with lack of epimerase activity causing the symptoms, they do not prove that this is the case. Nor is it evident why intracellular buildup of NADHX to the reported level of ∼5 pmol/mg protein [21], equivalent to ∼1 μM [22], would have such a severe impact in humans given that a similar accumulation in *Arabidopsis* had no discernable consequences [6] and that 1 μM NADHX corresponds to only ∼1% of the NADH pool in typical mammalian cells [23].

These considerations led us to use comparative genomic and genetic approaches to explore the possibility that NAD(P)HX epimerase has a moonlighting function. Our results build a reasonable circumstantial case that such a function exists, and that it involves B_6_ metabolism.

## Materials and methods

### Bioinformatics

DNA and protein sequences were taken from GenBank or SEED [24]. A set of >1,600 representative prokaryotic genomes was analyzed using SEED tools [24]. Full results of the analysis are encoded in the SEED subsystem named ‘NAD(P)HX epimerase–dehydratase’, which is publicly available at http://pubseed.theseed.org//SubsysEditor.cgi?page=ShowSpreadsheet&subsystem=NAD%28P%29HX_epimerase-dehydratase. Yeast coexpression data came from the SPELL database [25]. *Arabidopsis* coexpression data were analyzed using the ATTED database and its tools [26].

### Chemicals

NAD(P)HX was synthesized and purified as described previously [5,6]. L-Arabinose, potassium phosphates, LC-MS-grade water, acetonitrile, and isopropanol were from Fisher Scientific (Hampton, NH). All other chemicals were from Sigma Aldrich (St. Louis, MO).

### *E. coli* strains and expression constructs

The *yjeF* deletant was created by introducing the *yjeF* knockout allele from the Keio collection [27] into *E. coli* strain MG1655 by P1 transduction [28]. The kanamycin selection cassette was then excised [29]. To create the K192A mutant, the *yjeF* open reading frame with ∼500 bp of 5′- and 3′-flanking sequence was PCR-amplified (primers 5′-ggtacatgcatgcggaatatcgaccagcaattcgc-3′ and 5′-gctctagatctgatgcgatacatcatcccg-3′) from genomic DNA of *E. coli* strain MG1655, digested with SphI and XbaI, and ligated into pGEM4z to create *yjeF*500-pGEM4z. The conserved lysine 192 codon was changed to alanine with the QuikChange site-directed mutagenesis kit (Stratagene) using *yjeF*500-pGEM4z as template and primers 5′-ccatcacttttattgcgctggcaccaggcttgctcactgg-3′ and 5′-ccagtgagcaagcctggtgccagcgcaataaaagtgatgg-3′ to create *yjeF*500-K192A-pGEM4z. The K192A *yjeF* with flanking sequence was PCR amplified from *yjeF*500-K192A-pGEM4z (primers 5′-ggtacatgcatgcggaatatcgaccagcaattcgc-3′ and 5′-ggtacatgcatgctctgatgcgatacatcatcccg-3′), digested with SphI, and ligated into pCVD442 (which carries a β-lactam resistance marker) [30] to create *yjeF*500-K192A-pCVD442. The *yjeF*500-K192A-pCVD442 plasmid was transformed into a *yjeF* deletant whose kanamycin cassette had not been removed, and transformants were selected on LB medium containing 50 mg l^−1^ ampicillin. Merodiploids were identified by plating transformants on LB medium containing 10% (w/v) sucrose, and replated on LB-sucrose medium to obtain individual colonies. These colonies were then plated on LB alone or containing 50 mg l^−1^ of carbenicillin or kanamycin. Those that grew on LB but not on either antibiotic-supplemented plate were expected to have undergone the desired double recombination event to replace the kanamycin cassette with the *yjeF* K192A allele; this was confirmed by PCR. The *yjeF* open reading frame and flanking regions were sequenced to ensure that the strain was identical with wild-type except for the introduced K192A mutation.

For expression constructs, the *yjeF* or *yjeF* K192A open reading frame was amplified (primers 5′-ggaattccatatgaagaaaaaccccgtaagtatacc-3′ and 5′-ggaattctcagggagcggaattactcgattc-3′, pET28b or 5’-gggaattcatgaagaaaaaccccgtaagtatacc-3’ and 5’-gctctagatcagggagcggaattactcgattc-3’, pBAD24) from *E. coli* MG1655 genomic DNA or *yjeF*500-K192A-pGEM4z, digested with NdeI/EcoRI, and ligated into pET28b or pBAD24.

### Growth conditions

For metabolomics and NAD(P)HX analyses, six independent colonies of each bacterial strain were grown overnight in M9 minimal medium plus 0.2% (w/v) glucose and used to inoculate 2 ml (metabolomics) or 5 ml (NAD(P)HX analysis) of fresh medium to an optical density (600 nm) of 0.05. Cultures were grown at 42°C with shaking for 5–6 h until optical density reached 1.4  ±  0.1, then an equivalent of 2 ml culture (metabolomics) or 5 ml culture (NAD(P)HX analysis) at an optical density of 1.0 was harvested as previously described [31]. Cultures for assay of NADHX dehydratase activity were grown and harvested the same way except that culture volume was 50 ml.

For PLP analysis, bacterial strains were transformed with pBAD24 harboring pBAD24 alone or containing the *yjeF* open reading frame; fresh transformants were used to inoculate 50 ml of M9 minimal medium plus 0.5% (v/v) glycerol and 50 mg l^−1^ ampicillin; cultures were grown overnight and used to inoculate 1 L of fresh medium (also containing 0.2% (w/v) L-arabinose) to an optical density of 0.05. Cultures were grown at 37°C with shaking for 6–10 h until optical density reached 0.5  ±  0.05, then an equivalent of 10 ml culture at an optical density of 1.0 was harvested for total B_6_ vitamer analysis and the remaining culture was harvested by centrifugation for free PLP analysis.

For protein expression, *E. coli* strain BL21 (DE3) RIPL harboring a pET28b construct was grown in 200 ml of LB plus 50 mg l^−1^ kanamycin at 37°C until optical density reached 0.8. Cultures were then cooled to 22°C and isopropyl-β-3-D-thiogalactoside and ethanol were added (final concentration 0.5 mM and 4% (v/v) respectively). Incubation was continued overnight at 22°C and cells were harvested by centrifugation and stored at −80°C. Proteins were purified [5] and quantified [6] as described.

For assay of NADHX activity, cell pellets were resuspended in 1 ml of 25 mM Tris-HCl, pH 8.0, 300 mM KCl, 1 mM 2-mercaptoethanol on ice and sonicated (Fisher Scientific Ultrasonic Dismembrator, model 150E; 5 × 3 s pulses at 70% power, cooling on ice for 30–60 s between pulses). Lysates were centrifuged at 10000 ***g*** for 10 min; supernatants were desalted using PD MiniTrap G-25 columns (GE Healthcare) equilibrated with 25 mM Tris-HCl, pH 8.0, 100 mM KCl, 1 mM 2-mercaptoethanol).

### NADHX epimerase and dehydratase assays

Spectrophotometric assays were made at 22°C with a Beckman DU 7400 spectrophotometer, monitoring absorbance at 340 nm every 15 s. Assays (100 µl) contained 25 mM Tris-HCl, pH 8.0, 5 mM KCl, 2 mM MgCl_2_, 10 µg bovine serum albumin, 40 µM of a purified mixture of NADHX epimers, and 1 mM ADP. Reactions using purified proteins were started by adding 2 µg of either native or K192A YjeF protein. Two micrograms of *Arabidopsis* NAD(P)HX epimerase domain protein [6] was added at 4 min. NADHX dehydratase assays of cell lysates were started by adding 25 μl of desalted lysate.

### Metabolomics analyses

NAD(P)HX analysis was performed by liquid chromatography–mass spectrometry. Extraction and chromatographic separation were as described [6]. Cyclic NADHX was analyzed as two peaks [21] for which the data are reported separately. MS analysis was performed using a SCIEX 6500+ quadrupole linear ion trap equipped with a SCIEX Turbo Spray electrospray ionization source. Available standards were infused to obtain precursor/product transition ions for multiple reaction monitoring and to optimize acquisition parameters. The tripeptide Val-Tyr-Val was used as an internal standard for machine and injection control. Source and gas parameters were as follows: curtain gas, 25 psi; ion source gas 1, 40 psi; ion source gas 2, 50 psi; ion spray voltage, −4.5 kV; entrance potential, −4 V; temperature, 350°C. Metabolite-specific parameters are detailed in Supplementary Table S1. Untargeted metabolomics analysis was performed by gas chromatography–mass spectrometry as described [31]. BinBase numbers [32] are given for those metabolites that could not be positively identified. Raw data are available at metabolomicsworkbench.org (Study ID: ST000900). Metabolomics datasets were analyzed with ChemRICH chemical similarity enrichment software [33].

### Estimation of free PLP and total B_6_

To estimate PLP that is not tightly bound to large molecules (‘free’ PLP), pelleted cells (see above) were resuspended in 10 ml of Tris-HCl (pH 7.6) and broken by passage twice through a French Press at 2,000 psi. After centrifugation (3000 ***g***, 20 min, 4°C) to remove cell debris, the supernatant was ultracentrifuged (Beckman 70 TI rotor, 208000 ***g***, 1.75 h, 4°C) to pellet membranes. Protein concentration in the soluble fraction was determined with the Pierce BCA Protein Assay Kit (Thermo Scientific) using bovine serum albumin as standard, and adjusted to a final concentration of 6.33 mg/ml. Samples (4 ml) were passed through Amicon Ultra-4 3K centrifugal filter units (Millipore, 3 kDa cutoff) at 4°C, and ‘free’ PLP in the filtrate was estimated using a homogeneous enzymatic assay (A/C Diagnostics, San Diego, CA) [34]. Estimates used duplicate samples, each with two technical replicates. Total B_6_ extraction and analysis were performed as described [35] using triplicate samples. Free PLP and total B_6_ data were analyzed by one-way ANOVA.

## Results

### Comparative genomic analysis associates NAD(P)HX epimerase with vitamin B_6_

Over 1,600 representative prokaryote genomes were analyzed using the SEED comparative genomics database and its tools [24]; full results are available in the SEED subsystem named ‘NAD(P)HX epimerase–dehydratase’. The analysis detected 1,248 genomes with NAD(P)HX epimerase and dehydratase genes, 97% of which were fused together. The dehydratase occurred alone in 168 genomes, supporting the idea [1] that epimerase activity is dispensable, in some circumstances at least. The fused epimerase–dehydratase genes or standalone epimerase genes showed clustering associations with genes specifying the PLP-dependent enzymes alanine racemase and glutamate decarboxylase, and the PLP synthesis enzyme pyridoxine 5'-phosphate synthase (Figure 1B). Clustering with alanine racemase occurred in genomes from six phyla and clustering with pyridoxine 5'-phosphate synthase occurred in genomes from three phyla; clustering with glutamate decarboxylase was confined to Actinobacteria (Figure 1B). The taxonomic diversity of the organisms in which PLP-related genes cluster with epimerase–dehydratase genes makes the clustering association robust and implies a widespread functional relationship. As nearly all epimerase genes are fused to dehydratase genes, the clustering data do not show whether the PLP connection is with the epimerase, the dehydratase, or both. However, the occasional clustering of a standalone epimerase with alanine racemase (e.g. *Leuconostoc mesenteroides*, Figure 1B) is consistent with a PLP–epimerase connection.

Transcriptome data from yeast and *Arabidopsis* also support a B_6_ connection. Thus, in the yeast SPELL coexpression database [25] both the epimerase (*NNR1*) and the dehydratase (*NNR2*) correlate more strongly with the *GAD1* glutamate decarboxylase gene than with each other (Supplementary Figure S1A). Similarly, the ATTED plant coexpression database [26] shows the *Arabidopsis* epimerase–PPOX fusion gene to be embedded in a network of PLP-dependent enzymes and other genes of amino acid metabolism (Supplementary Figure S1B). Consistent with the reported lack of coexpression of the *Arabidopsis* dehydratase and epimerase–PPOX genes [7], the dehydratase is not in the epimerase–PPOX network (Supplementary Figure S1B). Nor does the dehydratase gene have a PLP-centric coexpression network of its own (Supplementary Figure S1B); this fits with the possibility that the PLP association in bacteria is with the epimerase, not the dehydratase.

### NAD(P)HX epimerase has a catalytically crucial lysine residue

**Figure 2:**
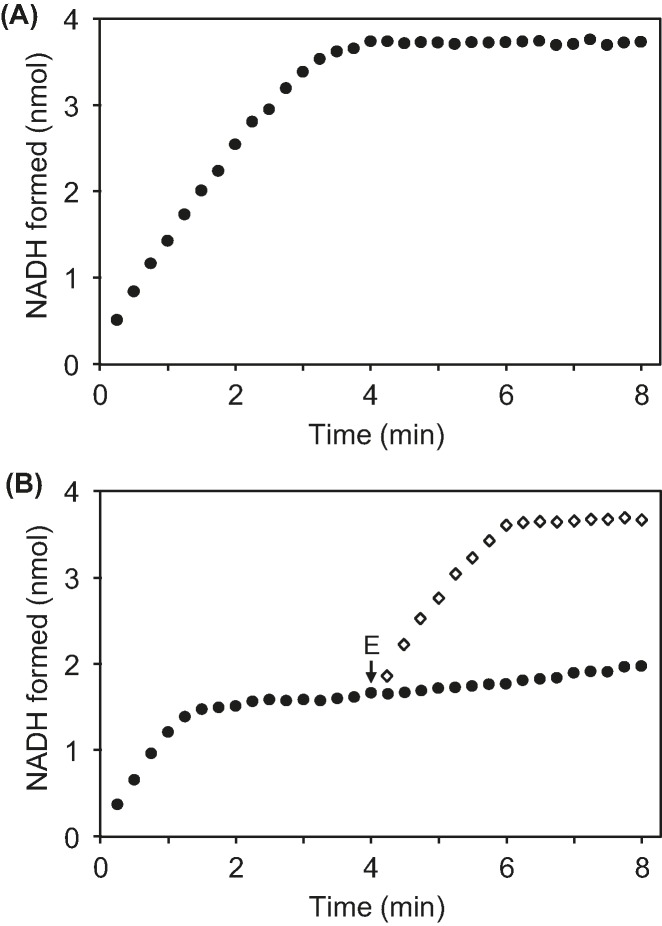
Activities of recombinant *E. coli* native and mutant (K192A) YjeF proteins (**A**) Assays (100 µl) contained 25 mM Tris-HCl, pH 8.0, 5 mM KCl, 2 mM MgCl_2_, 0.1 mg mL^−1^ BSA, 40 µM NADHX (containing approximately equal amounts of *S* and *R* forms), and 1 mM ADP. Reactions were started by adding 2 µg of native YjeF and absorbance was monitored at 340 nm at 22°C. (**B**) Assays were performed as above except that reactions were started by adding 2 µg of K192A YjeF (closed circles). In separate assays, 2 µg of *Arabidopsis* NAD(P)HX epimerase domain protein (E) was added at 4 min (open diamonds). Spontaneous epimerization of NAD(P)HX was undetectable in the conditions and time frame of the assay. Data are means of three replicates; S.E. was <0.17 nmol NADH formed for all data points.

Alignment of NAD(P)HX epimerase sequences from three domains of life revealed an invariant lysine about 45 residues from the C-terminus (Supplementary Figure S2). This lysine (K192 in *E. coli* YjeF) is located in the putative active site of the mouse epimerase crystal structure [11] and is thus an *a priori* candidate for a catalytic residue. We therefore used site-directed mutagenesis to replace K192 in *E. coli* YjeF with alanine, and used a coupled spectrophotometric assay [5,6] to compare the epimerase and dehydratase activities of the mutant and native proteins (Figure 2). The substrate was an NADHX preparation containing similar amounts of *S* and *R* forms. In the first phase of the assay, the rate of NADH formation measures conversion of the *S* form to NADH by dehydratase activity; this rate was the same for both proteins, confirming that the mutation does not affect dehydratase activity. As the *S* form is used up, the assay’s second phase measures epimerase-mediated conversion of the* R* form to the *S* form used by the dehydratase; this reaction rate fell by ≥95% in the mutant protein, demonstrating that epimerase activity is nearly abolished by the mutation.

### Ablating YjeF epimerase activity does not change total NAD(P)HX levels

**Figure 3:**
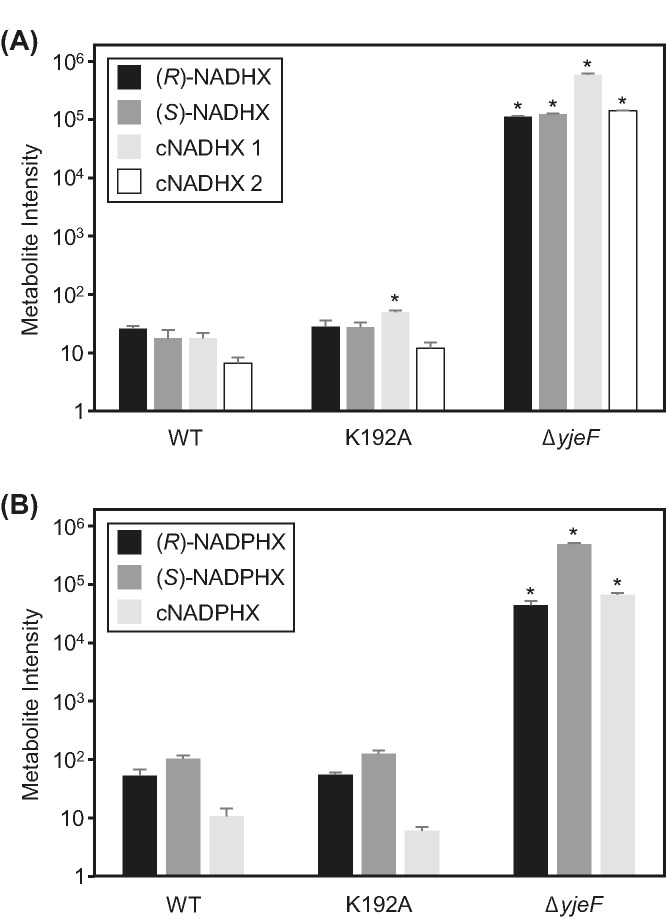
NAD(P)HX accumulation in *E. coli* wild-type, K192A, and Δ*yjeF* cells Cell extracts were analyzed by liquid chromatography–mass spectrometry with multiple reaction monitoring. Bars represent peak intensity of various forms of NADHX (**A**) or NADPHX (**B**). Data are means and S.E. of three replicates. Asterisks denote NADHX or NADPHX forms that are significantly different by ANOVA (*P*<0.05, Tukey HSD test) relative to wild-type. The data were log_10_ transformed for ANOVA and are plotted on a logarithmic scale.

Having a mutant YjeF protein with a specific and near-total deficiency of epimerase activity enabled us to probe the function of the epimerase protein in *E. coli* by replacing the native chromosomal *yjeF* gene with the K192A mutant gene. A *yjeF* deletant was used as a benchmark. Neither the K192A mutant nor the deletant showed obvious growth defects on rich (LB) or minimal (M9-glucose) media, as reported previously for the deletant [27], and NADHX dehydratase activity assays of cell extracts confirmed that the deletant lacked detectable activity whereas the K192A mutant and wild-type had similar activities (Supplementary Table S2). Targeted metabolomics analysis showed the expected large (three to five orders of magnitude) accumulations of *R*, *S*, and cyclic forms of NADHX and NADPHX in the deletant relative to wild-type (Figure 3A,B). In contrast, the K192A mutant had no significant increase in total NADHX or NADPHX, or in the levels of individual forms except for a slight rise in cyclic NADHX (Figure 3A). The K192A mutation’s lack of effects shows that ≥95% of YjeF epimerase activity is dispensable in the culture conditions used. Presumably spontaneous epimerization and/or residual epimerase activity (from the mutant YjeF enzyme or other, as-yet unidentified sources) effectively counter the accumulation of (*R*)-NAD(P)HX itself or its spontaneously cyclized derivatives in the K192A mutant.

### Ablating YjeF epimerase activity has unexpectedly large effects on the metabolome

**Figure 4:**
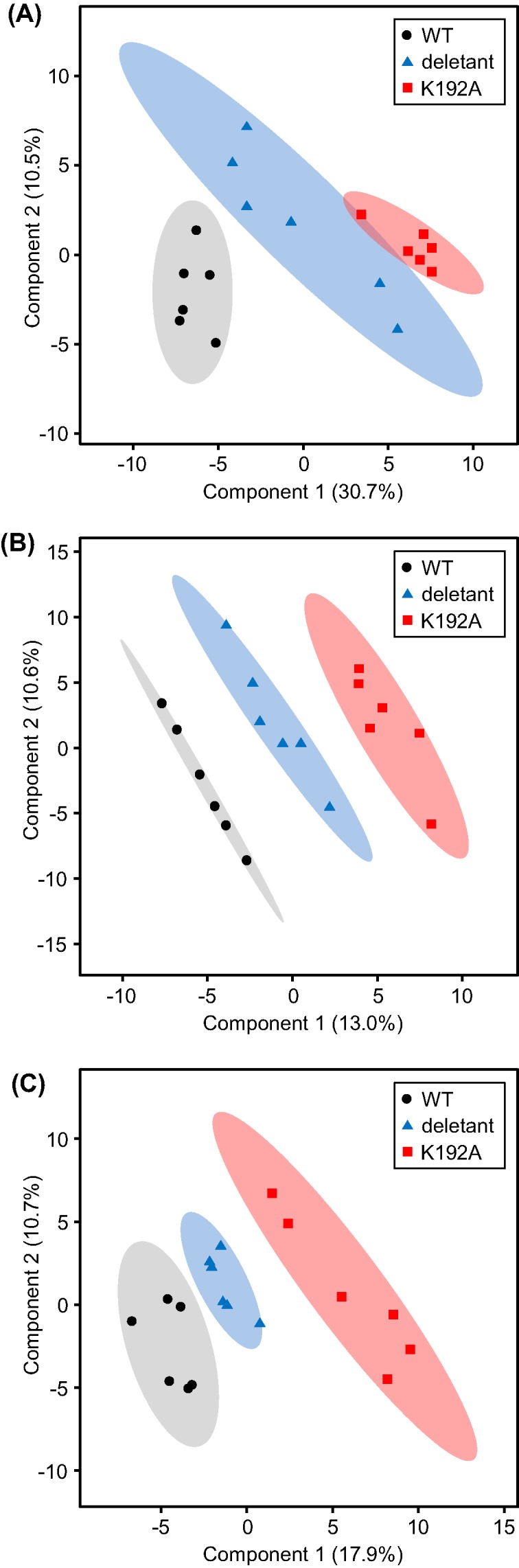
Partial least squares discriminant analysis (PLS-DA) of untargeted GC–TOF data PLS-DA analysis was performed on three independent metabolomics datasets (**A**–**C**) consisting of *E. coli* native (black circles), *yjeF* deletant (blue triangles), and K192A point mutant (red squares) cells. Gray, blue, and red ellipses display the 95% confidence interval of the respective data points.

**Figure 5:**
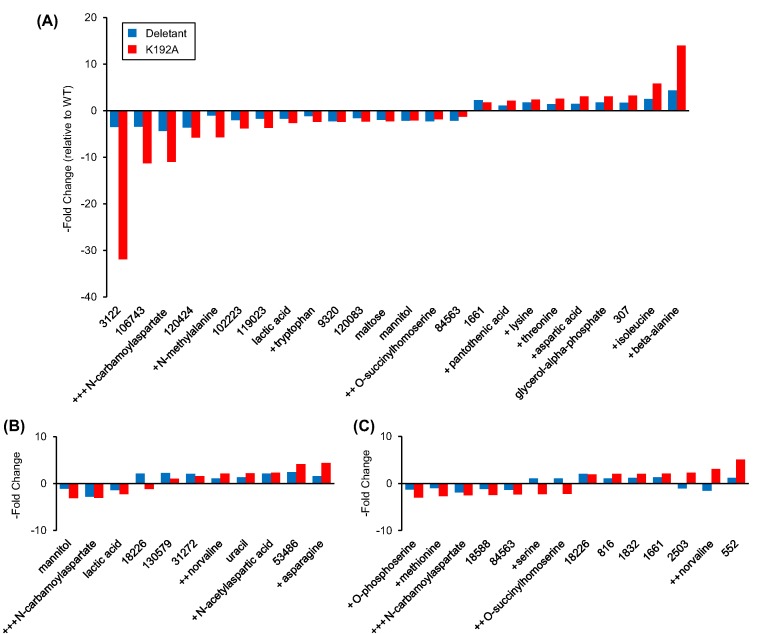
Metabolites that are present at different levels in *yjeF* deletant or K192A cells Metabolites whose levels changed significantly (*P*<0.05; *t* test) and 2-fold or more in *yjeF* deletant and/or K192A point mutant cells relative to wild-type are shown for three independent metabolomics datasets (**A**–**C**). Amino acids and related compounds are marked + if they appear in one dataset, ++ if they appear in two, and +++ if they appear in all three. The numbered metabolites are routinely observed in metabolomics datasets but have not been positively identified; numbers are BinBase identifiers, which are randomly assigned and do not reflect any structural information [32].

Given its lack of impact on NAD(P)HX pools, we expected the K192A mutation to have little or no effect on the metabolome as a whole. Surprisingly, however, the impact was large–in fact larger than that of deleting the entire *yjeF* gene encoding both epimerase and dehydratase activities. This paradox first became apparent in an exploratory survey of metabolome data from three independent experiments using partial least squares discriminant analysis (PLS-DA) [36]. In each dataset, the K192A mutant samples clustered far from the wild-type samples, with the *yjeF* deletant samples midway between them (Figure 4). The intermediate position of the deletant clusters indicated that the changes in individual metabolites in the deletant were broadly similar in nature to those in the K192A mutant but smaller in scale. Examination of individual metabolite peaks (identified and unidentified) confirmed that this was the case for all three experiments (Figure 5A–C). For metabolites that changed significantly relative to wild-type, the change was generally the same in direction (up or down) in both genotypes but greater in the K192A mutant.

Of the 155 metabolites identified in the three experiments, 69 changed significantly in at least one experiment; of these 69, over half (37) were amino acids or closely related metabolites. To further explore the impact of the K192A mutation, we performed chemical similarity enrichment analysis (ChemRICH) [33] on the three datasets. ChemRICH identifies highly impacted compound classes through the generation of metabolite clusters based on chemical similarity and ontology mapping. Amino acids were the only compound class significantly affected by the K192A mutation across all three experiments (Supplementary Figure S3), confirming the impact of the mutation on amino acid metabolism. Of particular note was the consistent behavior of the amino acid derivative *N*-carbamoylaspartate, an intermediate in pyrimidine biosynthesis. This metabolite was depleted in both the deletant and the K192A mutant in all three experiments (Figure 5A–C and Supplementary Figure S4).

**Figure 6:**
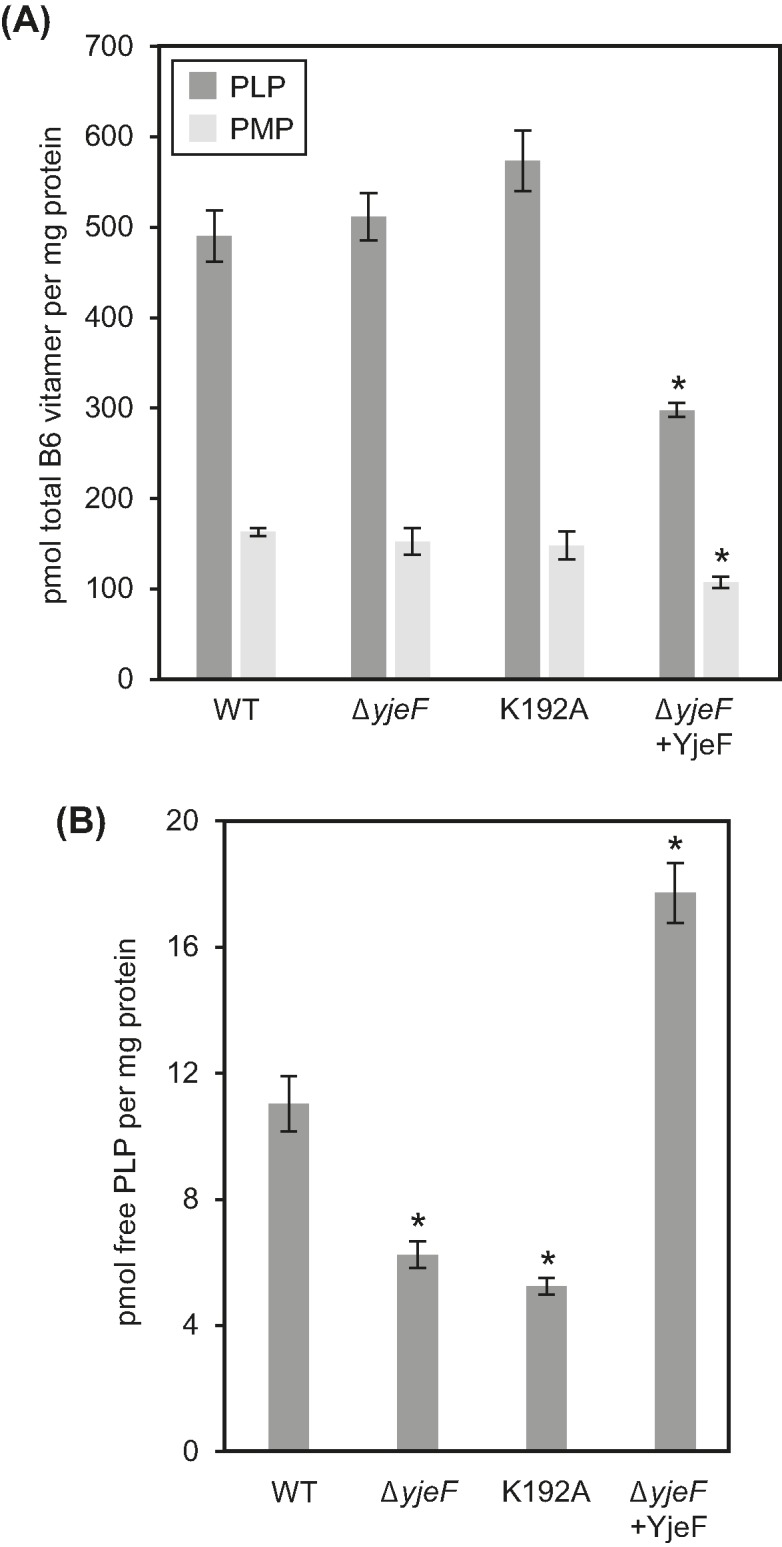
Vitamin B_6_ accumulation in *E. coli* wild-type, Δ*yjeF*, K192A, and YjeF overexpressing Δ*yjeF* cells (**A**) Total vitamin B_6_ levels. Data are means and S.E. of three replicates; PMP, pyridoxamine 5′-phosphate. Other B_6_ forms (pyridoxine 5′-phosphate, pyridoxal, pyridoxamine, and pyridoxine) were present in trace amounts below the limit of quantification. Data are means and S.E. of three replicates. Asterisks denote levels that are significantly different (*P*<0.05; ANOVA) relative to wild-type. YjeF overexpressing Δ*yjeF* cells grew ∼30% more slowly than wild-type cells under these conditions. (**B**) Free PLP levels. Data are means and S.E. of duplicates. Asterisks denote levels that are significantly different (*P*<0.05; ANOVA) relative to wild-type.

Given the centrality of PLP-enzymes to amino acid metabolism, these observations echo the epimerase–B_6_ connection inferred from comparative genomics (Figure 1B and Supplementary Figure S1B). For this reason, we measured the total pools of B_6_ vitamers (PLP, pyridoxamine 5′-phosphate, pyridoxine 5′-phosphate, pyridoxal, pyridoxamine, and pyridoxine) and estimated the pool of ‘free’ PLP using an enzymatic assay [34] that responds to the fraction of PLP that is not tightly protein-bound. While the ‘free’ PLP pool is only a small part of the total PLP pool, it is the form available to apo-B_6_ enzymes and hence crucial to PLP homeostasis and B_6_-enzyme activity [37]. Neither the K192A mutant strain nor the *yjeF* deletant had altered total B_6_ vitamer pools relative to wild-type cells (Figure 6A) but their ‘free’ PLP pools were significantly and similarly smaller (Figure 6B). Conversely, overexpression of YjeF in *yjeF* deletant cells significantly increased the size of the ‘free’ PLP pool (Figure 6B) even though the total B_6_ vitamer pools were reduced (Figure 6A).

## Discussion

The work we report here provides mutlifaceted circumstantial evidence that NAD(P)HX epimerase has a moonlighting function, and that this alternative function is in vitamin B_6_ metabolism. While the evidence comes from bacteria and plants, there is no reason to suppose that the epimerases from these organisms are the only ones that moonlight; mammalian and fungal epimerases may do so too. Moonlighting by the human epimerase NAXE would mean that the lethal consequences of *NAXE* defects [20,21] might not be due solely—or even at all—to NADHX accumulation.

Our evidence and arguments for moonlighting can be summarized as follows. (i) The fusion, clustering, and expression patterns of plant and prokaryote NAD(P)HX epimerase genes all point to an association with B_6_ enzymes. This cross-kingdom consistency is unlikely to result from chance alone. (ii) The K192A mutation in *E. coli* YjeF that almost eliminates epimerase activity has very little effect on NAD(P)HX pools but causes substantial changes in the pool sizes of other metabolites, particularly amino acids and ‘free’ PLP. As the latter changes cannot plausibly be attributed to effects of epimerase activity on NAD(P)HX levels, they are presumably due to a moonlighting activity of the epimerase protein that, in common with the epimerase activity, requires the conserved lysine residue.

The depletion of the ‘free’ PLP pool in the K192A mutant is expected to cause wide-ranging but differential reductions in the activities of B_6_ enzymes, which vary in PLP binding affinity and hence in susceptibility to PLP loss—and the attendant activity loss—when PLP becomes scarce [38,39]. It is therefore reasonable to invoke the smaller ‘free’ PLP pool as a cause of the scattered changes in the levels of amino acids and related metabolites in the K192A mutant (Figure 4). If reduced ‘free’ PLP pool size indeed underlies these metabolome changes, it would follow that the moonlighting function of the epimerase is in PLP homeostasis, for example, as a PLP chaperone. The depleted ‘free’ PLP pool in the *yjeF* deletant fits with an epimerase function in PLP homeostasis because deleting the epimerase or disabling it by the K192A mutation would be expected to have similar effects on ‘free’ PLP. The expanded ‘free’ PLP pool in cells overexpressing the whole YjeF epimerase–dehydratase protein is likewise consistent with the epimerase domain functioning in PLP homeostasis.

PLP homeostasis is critical because PLP is a reactive aldehyde that is toxic in the free (i.e. nonprotein-bound) form; this reactive PLP pool must therefore be held within limits that simultaneously avoid toxicity and meet the demands of B_6_ enzymes, of which *E. coli* has >50 [40]. Despite its criticality, the mechanism of PLP homeostasis is poorly understood both in terms of how PLP level is regulated and how PLP is delivered to the many target apo-B_6_ enzymes. Several proteins have, however, been implicated in the homeostatic mechanism. One of these is PPOX, which—in *E. coli* at least—has a noncatalytic PLP-binding site that may regulate free PLP concentration by acting as a PLP reservoir and channel PLP to apo-B_6_ enzymes [41]. Another potential player, from all kingdoms of life, is the COG0325 protein (YggS in *E. coli*, PROSC in humans), which also binds PLP [35,42]. Deleting *E. coli yggS* leads to complex amino acid-related growth phenotypes and to pleiotropic effects on the metabolome analogous to those found in the K192A mutant [35]. Purified recombinant NAD(P)HX epimerase proteins from *E. coli*, yeast, mouse, and plants are not reported to have the characteristic yellow color of B_6_ enzymes [5,6], suggesting that if they bind PLP, they do so too weakly for it to survive enzyme isolation. However, it should be noted that chaperones for other cofactors have diverse mechanisms that include folding the target protein into a conformation that favors cofactor binding and facilitating cofactor attachment as well as binding the cofactor and delivering it to the target [43]. Whatever the case, the link between the epimerase and PLP homeostasis indicates that vitamin B_6_ supplementation could, like niacin (vitamin B_3_) supplementation [21], be explored as a treatment strategy for the lethal epimerase deficiency disease in humans.

Our metabolic data have three particularly intriguing aspects. First is the lack of effect on NAD(P)HX pools of massively reducing the NAD(P)HX epimerase activity of YjeF (Figure 3A,B). This contrasts with a ∼20-fold accumulation of cyclic NADHX in epimerase-deficient human fibroblasts and smaller but significant increases in (*R*)- and (*S*)-NADPHX in these cells [21]. Possible explanations include differences between *E. coli* and fibroblasts in intracellular conditions such as pH [1] that affect the rate of spontaneous NAD(P)HX epimerization, or the presence of another—undiscovered—NAD(P)HX epimerase in *E. coli*. Residual epimerase activity (≤5%) in the K192A mutant enzyme is another possibility.

The second intriguing aspect is that the metabolomic effects of the K192A mutation were generally similar to—but larger than—those of the epimerase–dehydratase deletion even though the deletion caused massive buildup of NAD(P)HX and the K192A mutation did not. Deleting the dehydratase plus the epimerase thus partially reverted the effect of disabling the epimerase. We cannot yet propose an explanation for this enigma, but its existence reinforces our other evidence that the epimerase domains of bacterial YjeF proteins—and by extension, their homologs in other organisms—are more than just NAD(P)HX epimerases. If this is the case, it reinforces the need for caution in attributing the lethal effect of epimerase deficiency in humans [21] solely to NAD(P)HX accumulation.

The third intriguing aspect is the depletion of the pyrimidine precursor *N*-carbamoylaspartate in both the K192A mutant and the *yjeF* deletant in three independent experiments. This consistency, which is especially noteworthy given the typically high level of variability in untargeted metabolomics analyses of bacterial metabolites [44], suggests that the epimerase’s moonlighting function relates in some way to pyrimidine biosynthesis as well as to amino acid metabolism. The link could again be PLP. If the epimerase acts as a PLP chaperone, then deleting it or inactivating it by the K192A mutation would be predicted to drive up the concentration of PLP that is unbound to any protein *in vivo* and is thus truly free (as opposed to operationally ‘free’ *ex vivo*, as assessed by the enzymatic assay [34] we used). PLP is known both to inhibit the *E. coli* enzyme that produces *N*-carbamoylaspartate, aspartate carbamoyltransferase [45] and to react readily in physiological conditions with—and hence lower the concentration of—carbamoyl phosphate, the carbamoyl donor for this enzyme [46].

## Supporting information

**supplementary Figure 1:** Yeast and Arabidopsis transcriptomic evidence linking NAD(P)HX epimerase with PLP-dependent enzymes and amino acid metabolism. **(A)** Ranked lists of genes coexpressed with yeast NAD(P)HX epimerase (*NNR1*)and dehydratase (*NNR2*) genes from the SPELL database [25]. Each of these genes appears in the coexpression list of the other (black arrows), but at a substantially lower rank than the *GAD1* glutamate decarboxylase gene (reda rrows). ACS, Adjusted Correlation Score, a measure of weighted correlation for the gene with the query gene across all databases. **(B)** ATTED coexpression network of the *Arabidopsis* NAD(P)HX epimerase-PPOX gene (arrowed). The network includes five genes encoding PLP-dependent enzymes (circled in red) and five genes of amino acid metabolism (circled in blue). The network, built with the ATTED NetworkDrawer tool [26] was seeded using four PLP-related genes from the top 50 in the ATTED coexpressed gene list for the epimerase-PPOX gene (At5g49970). No such PLP-centric network was found for the NAD(P)HX dehydratase gene (At5g19150).

**supplementary Figure 2:** Alignment of NAD(P)HX epimerase sequences from diverse taxa. The sequences shown are of biochemically validated enzymes: human and mouse NAXE, *Arabidopsis* At5g49970, yeast NNR1, and *E. coli* YjeF. The predicted N-terminal targeting peptide regions are omitted for the mammalian and *Arabidopsis* sequences. Only the epimerase domains of the *Arabid-opsis* epimerase-PPOX fusion and the *E. coli* epimerase-dehydratase fusions are shown. Sequences were aligned with Multalin (http://multalin.toulouse.inra.fr/multalin/) and shaded with BoxShade (http://www.ch.embnet.org/software/BOX_form.html). Fully conserved residues are shaded black, fully conserved or conservatively replaced residues are shaded gray. The conserved, catalytically crucial lysine residue that was changed to alanine in *E. coli* YjeF is shaded red. The positions in human NAXE of the nonsense mutations (Tyr59*, Gln66*), the missense mutation (Asp218Val), and the deletions (Ala248Glufs*26, Lys270del) are marked †; all result in loss of function.

**supplementary Figure 3:** ChemRICH enrichment plots for metabolites altered in the K192A point mutant strain. ChemRICH enrichment results for metabolites whose levels changed significantly (*p*<0.05; *t* test) in K192A point mutant cells relative to wild type are shown for three independent metabolomics datasets (**A-C**). Circles represent clusters of related compounds. Circle sizes denote the number of metabolites in each cluster. Circle colors indicate an increase (red) or decrease (blue) in metabolite levels in K192A point mutant cells relative to wild type. The median XlogP (x-axis) represents the hydrophilicity of the cluster. The most significant clusters are at the top of the plot. Metabolites included in each cluster are as follows. AA: threonine, serine, *O*-succinylhomoserine, *O*-phosphoserine, norleucine, homoserine, glycine, β-alanine, alanine; AA basic: ornithine, lysine, glutamine, asparagine; AA acidic: *N*-carbamoylaspartate, *N*-acetylglutamate, *N*-acetylaspartic acid, glutamic acid, aspartic acid; BCAA: valine, norvaline, leucine, isoleucine, 2-aminobutyric acid; AA aromatic: tyrosine, tryptophan, phenylalanine; Hexoses: tagatose, methyl-*O-D*-galactopyranoside, mannose, levoglucosan, glucose, galactose, fructose, 6-deoxyglucose; Fructose (furanose) phosphates: ribose-5-phosphate, fructose-6-phosphate, fructose-1-phosphate, fructose-1,6-bisphosphate; Hexose phosphates: hexose-6-phosphate, glucose-6-phosphate, glucose-1-phosphate, galactose-6-phosphate; Alcohols: mannitol, arabitol, octadecanol.

**supplementary Figure 4:** Metabolites that changed significantly in at least two experiments. Metabolites whose levels changed significantly (*p* <0.05; *t* test) in *yjeF* deletant (KO) or K192A point mutant cells relative to wild type in at least two of three independent experiments (A-C) are listed. Boxes colored blue indicate a significant decrease in metabolite level relative to wild type, red boxes indicate a significant increase,and gray boxes indicateno significant difference. The numbered metabolites are routinely observed in metabolomics datasets but have not been positively identified; numbers are BinBase identifiers, which are randomly assigned and do not reflect any structural information [32].

**Supplemental Table S1:** Mass spectrometry multiple reaction monitoring (MRM) parameters for NAD(P)HX forms

**Supplemental Table S2:** NADHX dehydratase activity of cell lysates from wild type, K192A, and Δ*yjeF* cells. Activities were determined spectrophotometrically at 22°C. Values are means and S.E. of three replicates.

## Abbreviations

NAD(P)HX: NAD(P)H hydrate
PLP: pyridoxal 5′-phosphate
PPOX: pyridoxine/pyridoxamine phosphate oxidase
PLS-DA: partial least squares discriminant analysis
