# Supplementary material for: Evidence that the metabolite repair enzyme NAD(P)HX epimerase has a moonlighting function
Source: Biosci Rep. 2018 May 8;38(3):BSR20180223. doi: 10.1042/BSR20180223 (PMC5938422; doi:10.1042/BSR20180223)
Supplement: Supplementary file 1 [file bsr20180223_Supp1.pdf]

A

Epimerase *NNR1*

Rank	ACS	Gene
--	--	NNR1
1	2.8	GAD1
2	2.8	TFS1
3	2.7	DCS2
4	2.6	SOL4
5	2.5	GLK1
6	2.5	GTT1
7	2.5	PGM2
8	2.5	YJR096W
9	2.5	MSC1
10	2.5	PNC1
11	2.5	NNR2
12	2.4	ATG34
13	2.4	DCS1
14	2.4	BXI1
15	2.4	STF2
16	2.4	YBR139W
17	2.4	AIM19
18	2.4	GOR1
19	2.4	OM45
20	2.4	YNL115C

Dehydratase *NNR2*

Rank	ACS	Gene
--	--	NNR2
1	2.8	TFS1
2	2.7	GAD1
3	2.6	MSC1
4	2.6	DCS1
5	2.5	NTH1
6	2.5	YMR090W
7	2.5	YJR096W
8	2.5	PNC1
9	2.5	SOL4
10	2.5	NNR1
11	2.4	DCS2
12	2.4	GRE3
13	2.4	YMR196W
14	2.4	GLK1
15	2.4	PGM2
16	2.3	STF2
17	2.3	OM45
18	2.3	PRX1
19	2.3	RTN2
20	2.3	GLO1

B

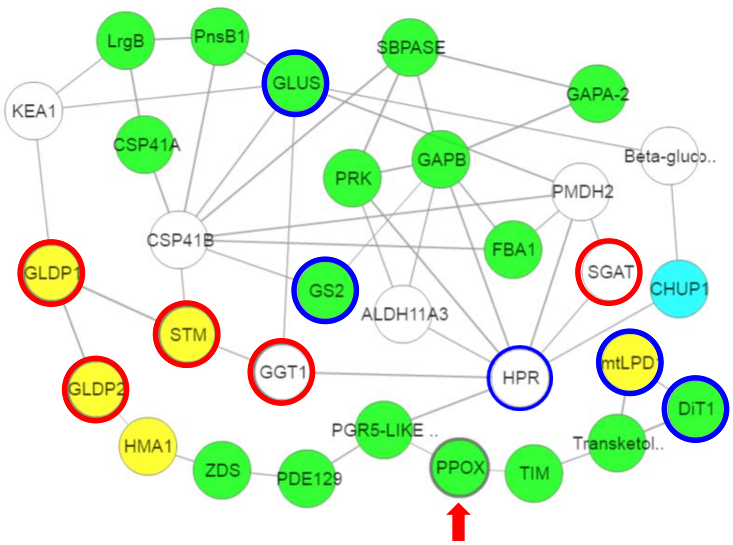

Supplementary Figure 1. Yeast and *Arabidopsis* transcriptomic evidence linking NAD(P)HX epimerase with PLP-dependent enzymes and amino acid metabolism.

(**A**) Ranked lists of genes coexpressed with yeast NAD(P)HX epimerase (*NNR1*) and dehydratase (*NNR2*) genes from the SPELL database [25]. Each of these genes appears in the coexpression list of the other (black arrows), but at a substantially lower rank than the *GAD1* glutamate decarboxylase gene (red arrows). ACS, Adjusted Correlation Score, a measure of weighted correlation for the gene with the query gene across all databases.

(**B**) ATTED coexpression network of the *Arabidopsis* NAD(P)HX epimerase-PPOX gene (arrowed). The network includes five genes encoding PLP-dependent enzymes (circled in red) and five genes of amino acid metabolism (circled in blue). The network, built with the ATTED NetworkDrawer tool [26] was seeded using four PLP-related genes from the top 50 in the ATTED coexpressed gene list for the epimerase-PPOX gene (At5g49970). No such PLP-centric network was found for the NAD(P)HX dehydratase gene (At5g19150).

```
                                †      †
Human_NAXE        48 DSEVMASTVVKYLSQEEAQAVDQELFNE-YQFSVDQLMELAGLSCATAIAKAYP--PTSMSRSPPTVLVICGPGNNGGDGLVCARHLKLF
Mouse_NAXE        42 GSETMAGAAVKYLSQEEAQAVDQELFNE-YQFSVDQLMELAGLSCATAIAKAYP--PTSMSKSPPTVLVICGPGNNGGDGLVCARHLKLF
At5g49970         64 NMQDSGSPPLSYLTQREAAEIDETLMGP-LGFSIDQLMELAGLSVAASIAEVYK--PEEYSR----VLAICGPGNNGGDGLVAARHLHHF
Yeast_NNR1         1       MSTLKVVSSKLAAEIDKELMGPQIGFTLQQLMELAGFSVAQAVCRQFPLRGKTETEKGKHVFVIAGPGNNGGDGLVCARHLKLF
E_coli_YjeF_N      1  MKKNPVSIPHTVWYADDIRRGEREAADVLGLTLYELMLRAGEAAFQVCRSAYP--------DARHWLVLCGHGNNGGDGYVVARLAKAV

Human_NAXE       135 GYEPTIYYPKRPNKP-LFTALVTQCQKMDIPFLGEMPA--EPMTIDELYELVVDAIFGFSFKGDVREPFHSILSVLKGLT---------V
Mouse_NAXE       129 GYQPTIYYPKRPNKP-LFTGLVTQCQKMDIPFLGEMPP--EPMMVDELYELVVDAIFGFSFKGDVREPFHSILSVLSGLT---------V
At5g49970        147 GYKPFICYPKRTAKP-LYTGLVTQLDSLSVPFVS-VED--LPDDLSKDFDVIVDAMFGFSFHGAPRPPFDDLIRRLVSLQNYEQTLQKHP
Yeast_NNR1        85 GYNPVVFYPKRSERTEFYKQLVHQLNFFKVPVLSQDEGNWLEYLKPEKTLCIVDAIFGFSFKPPMREPFKGIVEELCKVQNI-------I
E_coli_YjeF_N     82 GIEVTLLAQESDKPLPEEAALAREAWLNAGGEIHASN-----IVWPESVDLIVDALLGTGLRQAPRESISQLIDHANSHP---------A

                          †                               †                          †
Human_NAXE       213 PIASIDIPSGWDVEKGN--AGGIQPDLLISLTAPKKSATQFTGRY-----HYLGGRFVPPALEKKYQLNLPPYPDTECVYRLQ
Mouse_NAXE       207 PIASIDIPSGWDVEKGN--PSGIQPDLLISLTAPKKSATHFTGRY-----HYLGGRFVPPALEKKYQLNLPSYPDTECVYRLQ
At5g49970        233 VIVSVDIPSGWHVEEGDHEDGGIKPDMLVSLTAPKLCAKRFRGPH-----HFLGGRFVPPSVAEKYKLELPSYPGTSMCVRIG
Yeast_NNR1       168 PIVSVDVPTGWDVDKGPISQPSINPAVLVSLTVPKPCSSHIRENQTT---HYVGGRFIPRDFANKFGFEPFGYESTDQILKL-
E_coli_YjeF_N    158 PIVAVDIPSGLLAETGATPGAVINADHTITFIALKPGLLTGKARDVTGQLHFDSLGLDSWLAGQETKIQRFSAEQLSHWLKPR
```

Supplementary Figure 2. Alignment of NAD(P)HX epimerase sequences from diverse taxa. The sequences shown are of biochemically validated enzymes: human and mouse NAXE, *Arabidopsis* At5g49970, yeast NNR1, and *E. coli* YjeF. The predicted N-terminal targeting peptide regions are omitted for the mammalian and *Arabidopsis* sequences. Only the epimerase domains of the *Arabidopsis* epimerase-PPOX fusion and the *E. coli* epimerase-dehydratase fusions are shown. Sequences were aligned with Multalin (http://multalin.toulouse.inra.fr/multalin/) and shaded with BoxShade (http://www.ch.embnet.org/software/BOX_form.html). Fully conserved residues are shaded black, fully conserved or conservatively replaced residues are shaded gray. The conserved, catalytically crucial lysine residue that was changed to alanine in *E. coli* YjeF is shaded red. The positions in human NAXE of the nonsense mutations (Tyr59*, Gln66*), the missense mutation (Asp218Val), and the deletions (Ala248Glufs*26, Lys270del) are marked †; all result in loss of function.

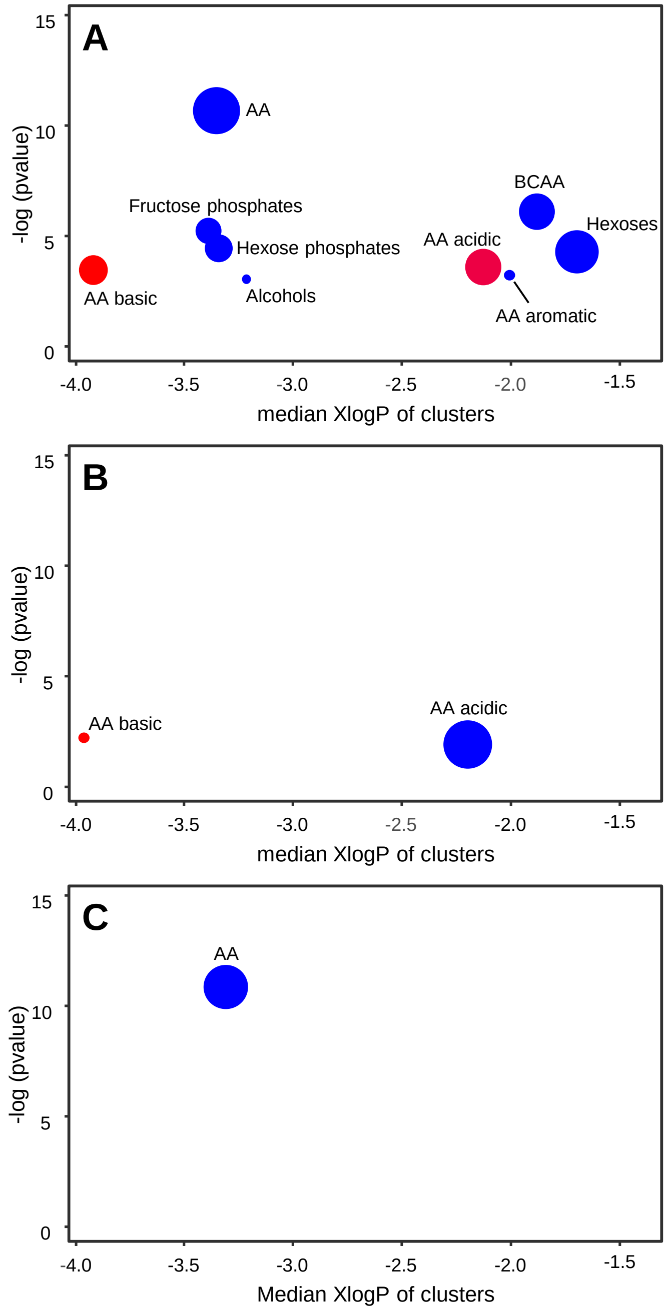

Supplementary Figure 3. ChemRICH enrichment plots for metabolites altered in the K192A point mutant strain.

ChemRICH enrichment results for metabolites whose levels changed significantly (*p* <0.05; *t* test) in K192A point mutant cells relative to wild type are shown for three independent metabolomics datasets (**A-C**). Circles represent clusters of related compounds. Circle sizes denote the number of metabolites in each cluster. Circle colors indicate an increase (red) or decrease (blue) in metabolite levels in K192A point mutant cells relative to wild type. The median XlogP (*x*-axis) represents the hydrophilicity of the cluster. The most significant clusters are at the top of the plot. Metabolites included in each cluster are as follows. AA: threonine, serine, *O*-succinylhomoserine, *O*-phosphoserine, norleucine, homoserine, glycine, ß-alanine, alanine; AA basic: ornithine, lysine, glutamine, asparagine; AA acidic: *N*-carbamoylaspartate, *N*-acetylglutamate, *N*-acetylaspartic acid, glutamic acid, aspartic acid; BCAA: valine, norvaline, leucine, isoleucine, 2-aminobutyric acid; AA aromatic: tyrosine, tryptophan, phenylalanine; Hexoses: tagatose, methyl-*O*-*D*-galactopyranoside, mannose, levoglucosan, glucose, galactose, fructose, 6-deoxyglucose; Fructose (furanose) phosphates: ribose-5-phosphate, fructose-6-phosphate, fructose-1-phosphate, fructose-1,6-bisphosphate; Hexose phosphates: hexose-6-phosphate, glucose-6-phosphate, glucose-1-phosphate, galactose-6-phosphate; Alcohols: mannitol, arabitol, octadecanol.

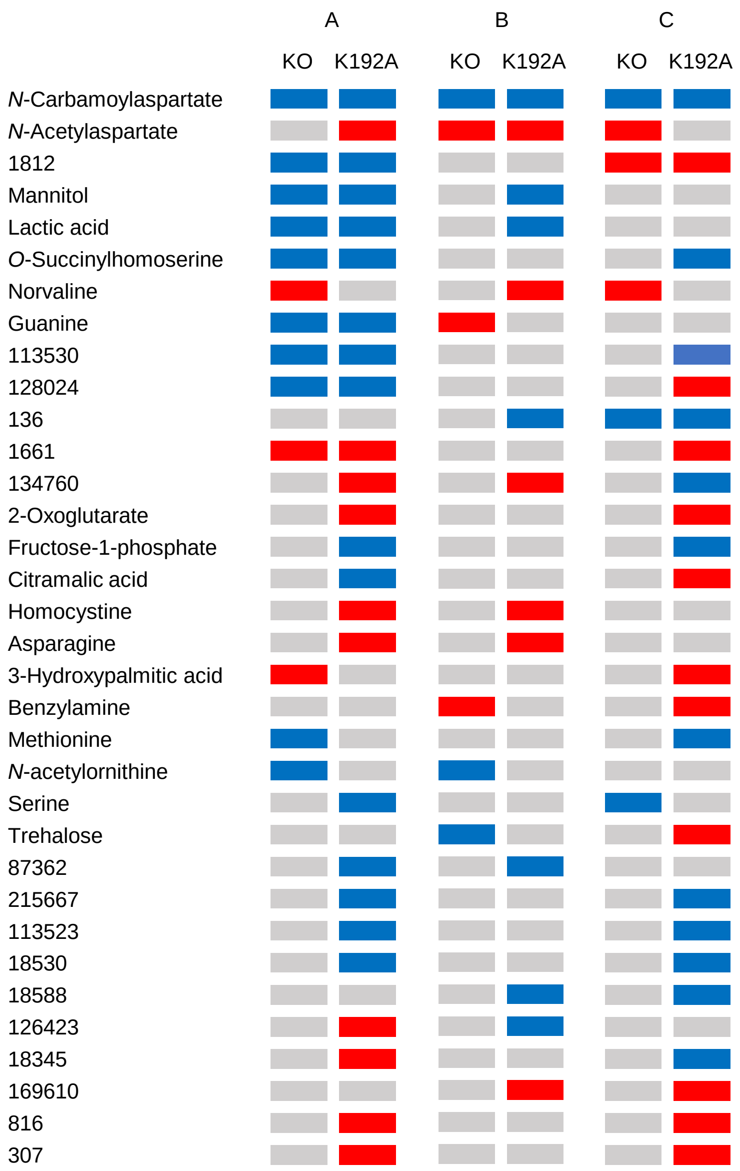

Supplementary Figure 4. Metabolites that changed significantly in at least two experiments.

Metabolites whose levels changed significantly (p <0.05; t test) in *yjeF* deletant (KO) or K192A point mutant cells relative to wild type in at least two of three independent experiments (A-C) are listed. Boxes colored blue indicate a significant decrease in metabolite level relative to wild type, red boxes indicate a significant increase, and gray boxes indicate no significant difference. The numbered metabolites are routinely observed in metabolomics datasets but have not been positively identified; numbers are BinBase identifiers, which are randomly assigned and do not reflect any structural information [32].

Supplementary Table 1 Mass spectrometry multiple reaction monitoring (MRM) parameters for NAD(P)HX forms

Period 1: 0.1 to 5.99 min

Metabolite	RT (min)	Q1	Q3	Scan time (msec)	DP (volts)	EP (volts)	CE (volts)	CXP (volts)
(S)-NADPHX quant	2.29	762.1	744.1	100	-100	-8	-35	-11
(S)-NADPHX qual	2.29	762.1	408.1	100	-150	-8	-50	-11
(S)-NADPHX $[M-2H]^-$ quant	2.29	380.5	200.8	70	-100	-8	-35	-11
(S)-NADPHX $[M-2H]^-$ qual	2.29	380.5	134.1	70	-10	-8	-35	-11
(R)-NADPHX quant	3.93	762.1	744.1	100	-100	-8	-35	-11
(R)-NADPHX qual	3.93	762.1	408.1	100	-150	-8	-50	-11
(R)-NADPHX $[M-2H]^-$ quant	3.93	380.5	200.8	70	-100	-8	-35	-11
(R)-NADPHX $[M-2H]^-$ qual	3.93	380.5	134.1	70	-10	-8	-35	-11

Period 2: 6.0 to 19.99 min

Metabolite	RT (min)	Q1	Q3	Scan time (msec)	DP (volts)	EP (volts)	CE (volts)	CXP (volts)
(S)-NADHX quant	8.60	682.1	346	100	-150	-10	-55	-11
(S)-NADHX qual	8.60	682.1	407.8	100	-150	-10	-50	-11
(R)-NADHX	10.98	682.1	346	100	-150	-10	-55	-11
cyclic NADPHX quant	7.52	744.0	408	100	-100	-8	-50	-11
cyclic NADPHX qual	7.52	744.0	426	100	-100	-8	-48	-11
cyclic NADHX 1 quant	14.50	663.9	396.8	100	-150	-10	-44	-11
cyclic NADHX 2 quant	15.59	663.9	396.8	100	-150	-10	-44	-11

Samples from experiment C were analyzed by LC-MS using targeted multiple reaction monitoring with two acquisition periods. Quant: quantitation ion; qual, qualification ion; RT, retention time; Q1, precursor ion m/z; Q3, product ion m/z; DP, declustering potential; EP, entrance potential; CE, collision energy; CXP, collision exit cell potential.

Supplementary Table 2. NADHX dehydratase activity of cell lysates from wild type, K192A, and Δ*yjeF* cells. Activities were determined spectrophotometrically at 22°C. Values are means and S.E. of three replicates.

Lysate Source	Activity (nmol min^{-1} mg^{-1})
Wild type	4.6 ± 0.5
K192A	3.1 ± 0.4
Δ*yjeF*	n.d.

n.d., not detected (detection limit: 0.1 nmol min^{-1} mg^{-1} protein).
